# Supplementary material for: Identification of Freezing-Responsive microRNAs and Their Targets in Chinese Jujube by Small RNA and Degradome Sequencing
Source: Int J Mol Sci. 2026 Sep 4;27(17):7912. doi: 10.3390/ijms27177912 (PMC13566890; doi:10.3390/ijms27177912)
Supplement: Supplementary file 1 [file ijms-27-07912-s001.zip › Supplementary Material S2/novel_miR_111.pdf]

Provisional ID : novel_miR_111
Score total : 4.1
Score for star read(s) : -1.3
Score for read counts : 0
Score for mfe : 3.8
Score for randfold : 1.6
Score for cons. seed :
Total read count : 287
Mature read count : 264
Loop read count : 23
Star read count : 0

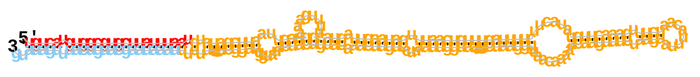

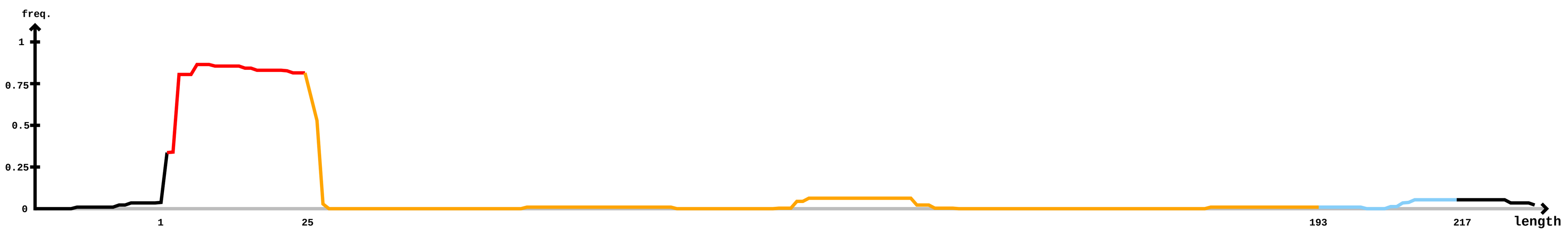

Mature Star

```
5'- cuugugguuagccacuaucaugucauguggcgugcuuauuuaauguauagcuuaauugaugaaaguuuugcaugauugaauaguuuaacuguuugauuuucuucauauguucaaaguuucuaacauuuaagaaucuuugaacaaaaacaaugggaaucaaaaaugguuaauuccuguucaaacaugucaucauaugguaggcuccaauucuuuaaauaagcaugucauuugauaugauagugguugauua -3'   exp
    ....((((((((((((((((((.(((((((((((((((((.(.((((((((..(((((.......(((((.((((((((.((((((((((((((((......((((((((((.((((.......)))).))))))))))........))))))))..)))))))))...))))))))..)))))))))).....))))))...)).).))))))))))))))))).)))))))))))))))))))))   reads   mm   sample
    .....gguuagccacuaucaugucaugu......................................................................................................................................................................................................................   3   0   LT-2
    ............cacuaucaugucauguggcgugc.......................................................................................................................................................................................................   4   0   JCK-2
    ..............cuaucaugucauguggcgu.........................................................................................................................................................................................................   4   0   LCK-3
    ...................augucauguggcgugcuuauu..................................................................................................................................................................................................   1   0   LT-1
    ....................ugucauguggcgugcuuauuu.................................................................................................................................................................................................   2   0   JCK-3
    ....................ugucauguggcgugcuuauuu.................................................................................................................................................................................................   2   0   JCK-1
    ....................ugucauguggcgugcuuauuuaau..............................................................................................................................................................................................   5   0   LT-3
    ....................ugucauguggcgugcuuCuuuaau..............................................................................................................................................................................................   1   1   LCK-3
    ....................ugucauguggcgugcuuauuuaau..............................................................................................................................................................................................   16   0   JCK-2
    ....................ugucauguggcgugcuuauuuaau..............................................................................................................................................................................................   7   0   JCK-1
    ....................ugucauguggcgugcuuauuuaau..............................................................................................................................................................................................   12   0   LT-2
    ....................ugucauguggcgugcuuauuuaau..............................................................................................................................................................................................   43   0   LCK-3
    ....................ugucauguggcgugcuuauuuaau..............................................................................................................................................................................................   2   0   LT-1
    ....................ugucauguggcgugcuuauuuaau..............................................................................................................................................................................................   5   0   JCK-3
    .....................Cucauguggcgugcuuauuuaaugu............................................................................................................................................................................................   1   1   LT-1
    ......................ucauguggcgugcuuauuuaaugu............................................................................................................................................................................................   17   0   JCK-1
    ......................ucauguggcgugcuuauuuaaugu............................................................................................................................................................................................   23   0   LCK-1
    ......................ucauguggcgugcuuCuuuaaugu............................................................................................................................................................................................   1   1   LCK-1
    ......................ucauguggcgugcuuauuuaaugu............................................................................................................................................................................................   19   0   JCK-2
    ......................ucauguggcgugcuuauuuaaugu............................................................................................................................................................................................   15   0   JCK-3
    ......................ucauguggcgugcGuauuuaaugu............................................................................................................................................................................................   1   1   LCK-1
    ......................ucauguggcgugcuuCuuuaaugu............................................................................................................................................................................................   1   1   LT-2
    ......................ucauguggcgugcuuauuuaaugu............................................................................................................................................................................................   9   0   JT-1
    ......................ucauguggcgugcuuauuuaaugu............................................................................................................................................................................................   19   0   LT-2
    ......................ucauguggcgugcuuauuuaaugu............................................................................................................................................................................................   5   0   LT-3
    ......................ucauguggcgugcuuauuuaaugu............................................................................................................................................................................................   11   0   LCK-3
    ......................ucauguggcgugcuuauuuaaugu............................................................................................................................................................................................   10   0   LCK-2
    ......................ucauguggcgugcuuCuuuaaugu............................................................................................................................................................................................   1   1   JCK-2
    ......................ucauguggcgugcuuauuuaaugu............................................................................................................................................................................................   7   0   LT-1
    ......................ucauguggcgugcuuauuuaauguU...........................................................................................................................................................................................   1   1   LT-1
    ......................ucauguggcgugcuuauuuaauguU...........................................................................................................................................................................................   1   1   LT-3
    ......................ucauguggcgugcuuauuuaauguU...........................................................................................................................................................................................   2   1   JCK-3
    ......................ucauguggcgugcuuauuuaauguU...........................................................................................................................................................................................   2   1   JCK-1
    ......................ucauguggcgugcuuauuuaauguU...........................................................................................................................................................................................   3   1   JCK-2
```

Mature Star

```
cuugugguuagccacuaucaugucauguggcgugcuuauuuaauguauagcuuaauugaugaaaguuuugcaugauugaauaguuuaacuguuugauuuucuucauauguucaaaguuucuaacauuuaagaaucuuugaacaaaaacaaugggaaucaaaaugguuaauuccuguucaaacaugucaucauaugguaggcuccaauucuuuaaauaagcaugucauuugauaugauagugguugauua
.........................uguggcgugcuuauuuaaugu...........................................................................................................................................................................................................   2   0   LCK-3
.........................uguggcgugcuuauuuaaugu...........................................................................................................................................................................................................   6   0   LCK-1
.........................uguggcgugcuuauuuaaugu...........................................................................................................................................................................................................   11  0   LCK-2
................................................................................uaguuuaacuguuugauuuucuuca................................................................................................................................................   2   0   JCK-2
................................................................................uaguuuaacuguuugauuuucuuca................................................................................................................................................   1   0   JCK-1
..........................................................................................................................acauuuaagaaucuuugaacaaaaacaauU.................................................................................................   1   1   LT-2
.............................................................................................................................uuuaagaaucuuugaacaaa........................................................................................................   6   0   JT-2
.............................................................................................................................uuuaagaaucuuugaacaaa........................................................................................................   7   0   JT-3
...............................................................................................................................uaagaaucuuugaacaaaaac.....................................................................................................   5   0   LT-2
...............................................................................................................................uCagaaucuuugaacaaaaac.....................................................................................................   1   1   LT-2
.................................................................................................................................................................................................ugguaggcuccaauucuuuaaauaag..............................   1   0   JCK-3
.................................................................................................................................................................................................ugguaggcuccaauucuuuaaauaag..............................   2   0   JCK-1
...............................................................................................................................................................................................................................ucauuugauaugauagugguugau..   3   0   LT-1
...............................................................................................................................................................................................................................ucauuugauaugauagugguugau..   1   0   LT-3
.................................................................................................................................................................................................................................auuugauaugauaguggu......   1   0   JCK-1
.................................................................................................................................................................................................................................auuugauaugauaguggu......   1   0   JCK-2
.................................................................................................................................................................................................................................auuugauaugauaguggu......   4   0   LCK-3
.................................................................................................................................................................................................................................auuugauaugauagugguugauu.   1   0   JCK-2
..................................................................................................................................................................................................................................uuugauaugauagugguugauu.   1   0   LT-3
...................................................................................................................................................................................................................................uugauaugauagugguugauua   5   0   LCK-3
```
